# Association between early diastolic dysfunction and increased peri-/epicardial fat: A CMR based study

**DOI:** 10.1186/1532-429X-18-S1-P111

**Published:** 2016-01-27

**Authors:** Darius Dabir, Rami Homsi, Daniel Kuetting, Julian A Luetkens, Christian Marx, Martin Sprinkart, Juergen Gieseke, Hans H Schild, Daniel K Thomas

**Affiliations:** Department of Radiology, University of Bonn, Bonn, Germany

## Background

Previous studies have shown that increased peri- and epicardial fat volume (PFV, EFV) leads to pathological diastolic strain (DS) and thus to cardiovascular morbidity. The aim of this study was to investigate the association between diastolic strain and EFV/PFV in obese and non-obese individuals without previous history of cardiovascular disease or additional cardiovascular risk factors.

## Methods

**Figure 1:**
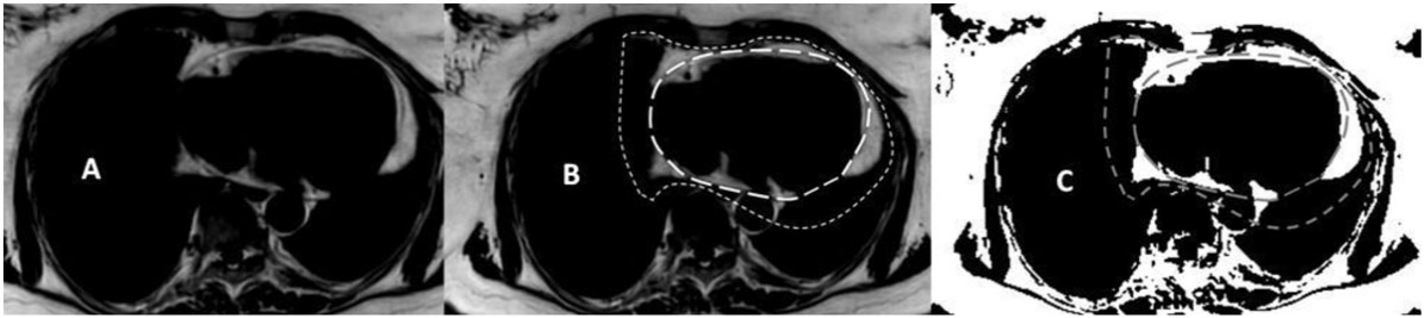
**Dixon image analysis for measurement of pericardial and epicardial fat volume (PFV, EFV) in a 60 year old health female**. **A.** Reconstructed fat only image. **B**. Reconstructed fat only image with corresponding regions of interest for EFV and PFV. **C.** Segemented fat voxels with transferred regions of interest.

19 healthy subjects (14 men, mean age 44.18 y ± 17.95) underwent a comprehensive cardiac magnetic resonance (CMR) examination (1.5 Tesla, Philips Ingenia). Patients were divided into two groups: (A) obese subjects with a body mass index (BMI) > 25 kg/m^2^; (B) non-obese subjects with a BMI <25 kg/m^2^. EFV and PFV were assessed using a 3D transversal ECG- and respiratory navigator gated mDixon-sequence (figure 1). FT derived systolic and diastolic circumferential strain parameters were calculated from SSFP-cine images in midventricular short axis, which were acquired prior to contrast agent injection (©TomTec, Image Arena).

## Results

Mean BMI in group A was 30.66 kg/m^2^ ± 5.71 and in group B 22.17 kg/m^2^ ± 1.76 (p < 0.05). Mean PFV and EFV were 238.61 ml ± 121.14 and 103.47 ml ± 37.05 in group A whereas mean PFV and EFV in group B accounted for 116.80 ml ± 43.14 and 60.71 ± 23.21 respectively (p < 0.05). Both groups revealed a peak circumferential systolic strain within a physiological range (group A:-23.60% ± 4.77; group B: -24.95% ± 3.52; p > 0.05). Group A (63.03 ± 11.83) showed significantly reduced mean early diastolic strain rates (EDSR) in comparison to group B (82.77 ± 16.33; p < 0.05). There were no significant differences between sex or age.

## Conclusions

Obese subjects with increased peri-/epicardial fat volume had a significantly reduced EDSR in comparison to the non-obese control group.

